# Nursing home staff’s views on residents’ dignity: a qualitative interview study

**DOI:** 10.1186/1472-6963-13-353

**Published:** 2013-09-16

**Authors:** Mariska G Oosterveld-Vlug, H Roeline W Pasman, Isis E van Gennip, Dick L Willems, Bregje D Onwuteaka-Philipsen

**Affiliations:** 1 Department of Public and Occupational Health, EMGO Institute for Health and Care Research, Expertise Center for Palliative Care, VU University Medical Center, Van der Boechorststraat 7, 1081 BT, Amsterdam, The Netherlands; 2 Department of General Practice, Section of Medical Ethics, Academic Medical Center, Amsterdam, The Netherlands

**Keywords:** Dignity, Elderly care physicians, End-of-life issues, Interviews, Nurses, Nursing home, Older people

## Abstract

**Background:**

Maintaining dignity is an important element of end-of-life care and also of the care given in nursing homes. Factors influencing personal dignity have been studied from both nursing home residents’ and staff’s perspective. Little is however known about the way nursing home staff perceive and promote the personal dignity of individual residents in daily practice, or about staff’s experiences with preserving dignity within the nursing home. The aim of this study is to gain more insight in this.

**Methods:**

A qualitative descriptive interview study was designed, in which in-depth interviews were performed with 13 physicians and 15 nurses. They expressed their views on the personal dignity of 30 recently admitted nursing home residents on the general medical wards of four nursing homes in The Netherlands. Interviews were transcribed and analyzed following the principles of thematic analysis.

**Results:**

According to both physicians and nurses, physical impairment and being dependent on others threatened the residents’ dignity. Whether or not this led to a violation of an individual resident’s dignity, depended - in staff’s opinion - on the resident’s ability to show resilience and to keep his/her individuality. Staff mentioned treating residents with respect and taking care of their privacy as most important elements of dignity-conserving care and strived to treat the residents as they would like to be treated themselves. They could often mention aspects that were important for a particular resident’s dignity. But, when asked what they could contribute to a particular resident’s dignity, they often mentioned general aspects of dignity-conserving care, which could apply to most nursing home residents. By attempting to give dignity-conserving care, physicians and nurses often experienced conflicting values in daily care and barriers caused by the lack of resources.

**Conclusions:**

Tailoring dignity-conserving care to an individual nursing home resident appears hard to bring about in daily practice. Both attention to solve contextual barriers within the nursing home as well as more awareness of staff members for their own values, which they take as a reference point in treating residents, is needed to promote personal dignity in the nursing home setting.

## Background

It is generally agreed that the maintenance of patient dignity is an important element in end-of-life care [1-4]. Within the context of care given at the end of life, the concept of dignity generally refers to *personal* dignity, a form of dignity which is individualistic and tied to personal goals and social circumstances. It is subjectively experienced, relates to a sense of worthiness, and can - as opposed to *basic* dignity - be affected by circumstances or the actions of others [5-7]. Several studies have investigated how patients’ personal dignity can be preserved or enhanced: by upholding a person’s autonomy [8] and by giving individualized care, restoring control, showing respect, performing advocacy and sensitive listening [9]. Also, finding out who the patients is as a person, what is important to them, and what they value has been reported to be fundamental for care that may conserve or bolster the dignity of patients nearing death [5,10].

Although some core elements of promoting dignity have been found to apply across all care settings, it has been mentioned that nurses should identify and address dignity issues specific to their own practice areas [11]. A setting where giving dignity-conserving care is of great significance, is the nursing home. Along with the ageing population, nursing homes have increasingly become a place where many older people with multi complex needs are cared for until death [12,13]. Nursing home residents are a vulnerable group with regard to loss of dignity, not only because they are functionally incapacitated and need care, but also because they increasingly lack social networks [14,15]. Factors influencing personal dignity in the nursing home have been explored in some studies; both from the residents’ perspective [14-17] as well as from the perspective of nursing home staff [18,19]. The two last-mentioned studies mainly focussed on staff’s perceptions on their own dignity while working in the nursing home and revealed threats to dignity caused by ethically difficult situations and moral conflicts created by lack of time and resources in the nursing home.

Little is however known about the way nursing home staff perceive the personal dignity of residents, or about their efforts to enhance residents’ dignity within the nursing home. One of the few studies on this subject found that older persons’ dignity was promoted by providing for the person’s physical needs, and respecting one’s identity and integrity [18]. The conclusion of a study in the United Kingdom was that, despite stated intentions to promote dignity, the circumstances of some elderly care institutions rendered this impossible for staff, and caused patients to experience avoidable indignities [20].

Furthermore, considering the weight given to the concept of individualized care in preserving dignity, it is not only relevant to investigate nursing home staff’s views on residents’ dignity in general, but also to become more specific and investigate to what extent nursing home staff members give dignity-conserving care that is tailored to an individual nursing home resident. To this end, the following research question was formulated: How do nursing home staff view and promote the personal dignity of individual nursing home residents in daily practice, and what are staff’s experiences with preserving dignity in the nursing home?

## Methods

### Design

We conducted a qualitative descriptive study [21], in which in-depth interviews with physicians and nurses working in a nursing home were performed.

### Study population and recruitment

The recruitment of physicians and nurses followed on the participation of nursing home residents in an earlier phase of this study, in which 30 recently admitted nursing home residents on the general medical wards (long-stay units for people with severe physical illnesses) of four nursing homes in The Netherlands were interviewed about factors that influenced their personal dignity, either in a positive or negative way. These residents ranged in age between 49 and 102 years and suffered from a variety of diseases. Two residents were terminally ill, and all others experienced deterioration of bodily functions which disabled them in such a way that they needed plural, complex continuing care and monitoring. Details about the recruitment, inclusion criteria and characteristics of these residents are shown in the Appendix.

By participating in the study, these residents gave permission to ask both their physician and primary attending nurse about their view on the personal dignity of the resident concerned. Because physicians and nurses generally cared for more than one of the participating residents, 13 physicians and 15 nurses were eventually identified to be responsible for these 30 residents and were asked to express their view on the personal dignity of the resident(s) they cared for. They were all willing to participate.

Table 1 presents the variation within the groups of these physicians and nurses in terms of gender, age, education, work experience and cultural background. The 13 physicians included nine women and four men and were all employed within the nursing home. Most of them were elderly care physicians, whereas others were still in training, General Practitioner or Medical Doctor. All but one of the 15 nurses were women. This group mainly consisted of Certified Nursing Assistants, and a few Registered Nurses. The majority of physicians and nurses had many years of work experience in the nursing home setting.

**Table 1:** Characteristics of the participants

		**Physicians (n=13)**	**Nurses (n=15)**
**Gender**	Female	9	14
	Male	4	1
**Age range**	25–34	3	4
	35–44	5	3
	45–54	2	6
	≥ 55	3	2
**Education**	Elderly care physician	8	-
	Elderly care physician (in training)	1	-
	General practitioner	1	-
	Medical doctor	3	-
	Certified nursing assistant	-	12
	Registered nurse	-	3
**Years of experience in nursing home care**	1–5	3	2
	> 5	10	13
**Cultural background**	Dutch	13	7
	Surinam	-	5
	Other	-	3

### Data collection

In-depth interviews with physicians and nurses were conducted from July 2010 to August 2011 and followed shortly after the interview held with a particular resident. The interviews were guided by a topic list and consisted of a general part, in which the interviewees were questioned about their view on and experiences with dignity and dignified care in their daily work in the nursing home, and a larger more specific part, in which they expressed their view on the personal dignity of a particular resident. Questions were not asked in an established order, but followed up on the answers nursing home staff provided. All interviews were performed by the first author, took place in the nursing homes and were recorded and transcribed verbatim. The interviews lasted approximately 30 minutes.

### Data analysis

Data analysis started during data collection and was an ongoing process. We coded and analyzed the transcripts with the aid of Atlas-ti software, following the principles of thematic analysis [22]. The transcripts were first read and re-read to become familiar with the data. Then, codes (e.g. limited space, acceptance, taking someone seriously) were ascribed to meaningful text units. After coding several interviews in this inductive manner, the research group discussed the evolving code list and compared the codes with the content of the interview transcripts. To ensure reliability of the coding procedure, several interviews were coded independently by the first and third author by using the same evolving code list, which revealed high consensus. Any difficulties were discussed with the other authors, all experienced in performing qualitative research. Codes were grouped together, and we searched for themes among them. Four main themes were identified: residents’ ability to keep their individuality, treat others as one would like others to treat oneself, general dignity-conserving care for individual nursing home residents, and conflicting values with regard to dignity in daily care. Relevant quotes were chosen to illustrate these themes.

### Ethical considerations

Our study was approved by the Medical Ethics Committee of the VU University Medical Center, Amsterdam. The Management Teams and Clients’ Councils in the nursing homes gave their approval for the research to be carried out. Both residents and nursing home staff gave their informed consent before each interview. Aspects that came up in the residents’ interviews were not shared with physicians or nurses.

## Results

### Residents’ ability to keep their individuality

Although several physicians and nurses indicated that ‘dignity’ was not a word they used in ordinary daily language, they were nevertheless able to reflect on what it meant to them in daily practice. When they were asked about their view on residents’ dignity, it appeared that many nursing home staff members considered someone’s level of dignity to be interwoven with his/her character and attitude to life. In assessing whether or not a resident felt dignified they often took his/her personality traits, cognitive abilities, resiliency and appearance into account:

Lots of people have had a bad experience or have been ill and are basically in a terrible situation because they are no longer able to cope at all and have become dependent. But they still manage to make something of the situation simply because they are mentally strong. They are optimistic, they look good, and they still take good care of themselves. And I do think that is dignity, a kind of dignity. (Elderly care physician, 35–40 years)

Both physicians and nurses indicated that being functionally incapacitated and, consequently, dependent on others threatened the personal dignity of nearly all nursing home residents. They stated that residents who had suddenly lost independency had a harder time to accept their admission to a nursing home than residents who had progressively become more dependent. Nevertheless, nursing home staff said to observe that a lot of residents could adjust to their new situation over the course of time; from difficulties with settling down in the beginning to accepting and living a satisfying life later on. If residents however lost their individuality and got hospitalised - i.e. becoming bound up in the daily rhythm of the nursing home in such a way that someone forgets to think him/herself - staff evaluated this as undesirable and undignified:

You notice that people get hospitalised very quickly. They leave an awful lot of decisions to you, because “you know so much better, Sister”. And I think that’s a kind of loss of your own identity, your own dignity. . . . If I was in that position, I would find it really awful if I was to start behaving in that dependent way - I would no longer be me. (Certified Nursing Assistant, 45–50 years)

Despite their observations that residents often adjusted to their new situation over the course of time, which could restore residents’ sense of dignity, both physicians and nurses indicated they hoped to never end up in a nursing home in the future themselves. In addition to the undermining influence of losing independency, nursing home staff members ascribed the violation of dignity to the lack of space, personal belongings and resources in the nursing home, i.e. the staffing shortages, and consequently the lack of time and attention that residents could receive. Violation of dignity within the nursing home was, according to staff, most present when residents had to wait for help when they urgently needed to go to the toilet, and when too late, needed help with changing clothes. Most staff members thought that more money (spent to recruit more personnel) would be the only solution. Some physicians however mentioned that more training for nurses (e.g. training to be a good hostess) and more work efficiency would be part of the solution toward a nursing home environment that conserved the individuality and personal dignity of residents:

I think that when it comes to a point that you have so little time, and it becomes routine, that routine is simply deadly. That’s also because it stops you seeing that you are dealing with people. There comes a point when everything has to be done so quickly, one thing after another, that sometimes you no longer realise “Darn, I am dealing with a real human being here”. . . . And I also think that by doing things in a more dignified way, doing it more calmly and taking more time, in other words, that you might even get finished more quickly in the end. There is much less of a risk that you might have to go back. For example “darn, I forgot the teeth”, or “darn, she’s still wearing her glasses”, that kind of thing. And I also think that if you give people the chance to say how they are feeling, you are also find that people are less likely to press that bell. . . . Yes, just giving people a little bit of attention every day will cost you less time than clearing up all the mess when it finally gets out of hand. (Elderly care physician in training, 25–30 years)

### Treat others as one would like others to treat oneself

Both physicians and nurses stated that maintaining residents’ dignity was important to them. In their efforts to maintain or promote a resident’s dignity, several physicians and nurses mentioned that they intended to proceed from the concept ‘treat others as one would like others to treat oneself’, thereby taking their own preferences and values into account and projecting these on the care situation:

Treating other people as you yourself would want to be treated: that is the key feature of dignity for me. . . . respectful, that is definitely the most important thing I think, a respectful approach and treatment. (Certified Nursing Assistant, 25–30 years)

You can't just leave somebody naked halfway through, no that’s not nice. I wouldn't want that to happen to me. So what we try and do is to say that anything you wouldn't like yourself, you mustn't do to the people here. (Registered Nurse, 45–50 years)

For both physicians and nurses one of the core elements of caring with dignity was treating residents with respect. This entailed caring for a resident according to his/her wishes, caring with attention, having a little chat while washing or dressing the resident and taking the resident seriously. Also taking care of someone’s privacy was frequently mentioned as a dignity-conserving aspect of nursing home care. Three forms of privacy could thereby be distinguished: environmental privacy (e.g. knocking on someone’s door before entering the room), physical privacy (e.g. covering the resident when laying naked when other nurses enter the room) and confidentiality (e.g. no communication about a residents’ health status when other residents are near).

Having respect for privacy, you know - sufficient peace, space if you are busy with someone, not being disturbed, listening to them, as well as giving them enough information. And reaching good decisions together, where you take your patients' wishes into account, but also where you explain clearly what would be a smart move and what wouldn’t. And where you are able to come to a good policy together and actually carry it out. (Elderly care physician, 35–40 years)

I think you should do everything you can to safeguard privacy. So if you are busy washing someone, keep the door closed. And when you come in you cover them with a sheet. That’s also a question of dignity. And calling people Mr or Mrs, treating them decently. Yes, what I mean here is that you listen to people properly. That you observe people properly and try to interpret what they mean. Also take care in how you respond to the story they are telling. So don't make a fool of people. (Certified Nursing Assistant, 60–65 years)

In addition, inherent to their responsibility for the residents’ medical treatment, physicians were more concerned than nurses about a resident’s functional abilities and thought more about ways to increase residents’ independency, for instance by providing the resident with an electric wheelchair. Nurses however reported more concerns about the quality of the daily care as aspects influencing dignity, e.g. the frequency with which residents could be showered and the notion that some residents felt a burden to the busy nurses.

### General dignity-conserving care for individual nursing home residents

Nurses and physicians could often mention some aspects that were relevant for the personal dignity of an individual nursing home resident, e.g. receiving social support of a certain family member, reading books, participating in organized activities, or wearing nice clothes. However, despite this knowledge, when physicians and nurses were asked what they could do to preserve or support a particular resident’s dignity, the majority of them came up with the same elements important for dignity-conserving care as they had already reflected on in the more general part of the interview and which could apply to most nursing home residents:

Interviewer: And what would be important things that the carers, or you, could do to keep his dignity?

Respondent: Specifically, just for him? Well, I think it's always a question of giving somebody attention and taking somebody seriously. Behave nicely to someone, respond to what someone says. . . . Really, I think it's mainly the kind of things that apply to anyone. (Elderly care physician, 60–65 years)

A minority of physicians and nurses were more keen in getting to know the background of a nursing home resident. They tried to stimulate a resident in continuing activities he/she used to perform, and paid attention to the things that characterized a particular resident, with the ultimate goal to continue a resident’s self and identity:

She is also quite interested in her appearance with her lipstick and her jewellery. . . . Yes, she does find personal care important. . . . So dignified care is when you have that lipstick, you make sure that she puts it on. And that perfume, you know, that she doesn't… that she can't be bothered any more, saying “that lipstick and going to the hairdressers’, I don't need that any more”. That she still feels she has her dignity, that you pay attention to that, because it often happens very gradually, you know, I won't put on any perfume anymore, and the next step is well you know that lipstick, it's not so important. . . . Yes, well we're not able to put in the curlers every day, unfortunately, but you do try to do that kind of thing as much as possible. (Registered Nurse, 30–35 years)

To help a resident to preserve his/her sense of self and personal identity, the resident’s habits, preferences and hobbies were questioned and written down at the time of nursing home admission. Some staff members mentioned that these written down aspects were even looked at once in a while. Nevertheless, because of scarcity of personnel, tailoring activities to individual residents appeared to be difficult:

People are asked what they used to like doing. It is part of the intake form. I think nothing is done with this information an awful lot of the time, simply because of a shortage of staff. I think the relatives often have to take the initiative. And the activities staff could do things. They basically organise a few general activities for everyone, but I think they don't have enough people to be able to offer really personalised things. (Medical Doctor, 25–30 years)

### Conflicting values with regard to promoting dignity in daily care

Nursing home staff members gave examples of situations in which they tried to prevent the violation of a resident’s dignity, even when the resident was not aware of his/her dignity being threatened. In doing this, staff sometimes experienced conflicting values themselves:

Yes, of course you do have a position of power in the health service, and of course you mustn’t abuse that position. But you can, he's in a wheelchair, so you say “I'll just take you back to your room”, even though he doesn't want to go. You do have to do that. I mean, I can hardly have him getting undressed in the sitting room. . . . Yes, and it doesn't always feel right, because you still have the feeling that you are abusing your power or something, but you have to keep in the back of your mind that you are doing it to protect him. The end justifies the means, as it were. But that is quite difficult, because you still have the feeling inside that you would really rather not be doing it - but sometimes you just have to, don't you? (Certified Nursing Assistant, 40–45 years)

Dilemmas were also experienced when ‘respecting a resident’s wishes’ meant that other residents’ dignity could be violated, e.g. if a resident’s behaviour caused other residents to feel uncomfortable (as might arise from the situation described in the quote above), if a resident interfered too much in other’s lives, or if a resident had developed strong body odour. In addition, nursing home staff members said to struggle in situations in which a resident wanted to stay in bed all day despite having enough energy to get up. Again, this created a conflict for them. Physicians and nurses always tried to stimulate a resident to get out of bed, but indicated that the resident eventually decided. As shown in the following example, staff members tried to come to a compromise, to create a situation that was dignified for the resident and compatible to their own professional attitude:

We let him keep his dignity and let him do things as he wants, but we do impose our own values a little bit because we go and check up on him every two hours. You could also say, let's just leave him. But you also have your professional attitude: you don't leave him lying there just because you know he is not asking for anything. So yes, that is the agreement you make with each other. . . . You really do have to check up on him every three hours, if you don't do that you could easily find that he has given up the ghost. (Certified Nursing Assistant, 50–55 years)

When asked about undignified situations in the nursing home, especially nurses mentioned situations that had to do with the limited capacity of caring personnel, i.e. the lack of time and attention that they could spent on residents. They indicated that paperwork had increasingly become part of their job and said that they often would like to provide more in the care of older people than they were able to deliver. This could not only hamper the dignity of residents, but also that of their own, and created an inner conflict:

Yes, we just don't have enough money, or enough time. That is the lack of dignity, you have to do everything so quickly, that's what I find really undignified. . . . And what we could do more of on the wards is palliative care, making sure that we really do have extra staff for that. . . . Sometimes you have two people in the final stage and you are there doing the night shift on your own. Well, that’s not nice. You feel… simply… you go home feeling terrible because you just want to be able to sit with someone if they are frightened. Of course you check up on them every 15 minutes anyway. Then you are lucky if you have had a quiet night, because if you've had a busy night, well, then you just feel miserable . . . .because of course the residents are the priority, but the staff are important too of course. The staff need to be able to enjoy their work. Dignity also applies to the staff. You need to be able to keep your dignity too. And that is often rather difficult, we get decrees from above and then we have to straighten things out for the residents, and you don't always have a good feeling when doing this. (Certified Nursing Assistant, 45–50 years)

## Discussion

This study investigated the views and experiences of nursing home staff members regarding the personal dignity of Dutch nursing home residents on general medical wards. According to physicians and nurses, the most important elements of dignity-conserving care were treating residents with respect and safeguarding their privacy. Also enhancing a resident’s autonomy and the ability of a resident to keep his/her individuality were seen as important. In contrast to the latter, staff members mentioned general themes of dignity-conserving care when asked how they promoted an individual residents’ dignity. By attempting to give dignity-conserving care, staff members strived to treat the residents as they would like to be treated themselves and often experienced conflicting values and barriers related to the nursing home context.

### Similar perceptions of nurses and physicians on residents’ dignity

In exploring the experiences and views of both physicians and nurses, we hardly found any differences in their perspectives on an individual residents’ dignity and elements that conserve dignity. Both were concerned with physical as well as psychosocial aspects influencing dignity. Earlier research in other contexts showed that physicians mainly focussed on physical aspects of suffering and less on psychosocial aspects [23,24]. The way in which the Dutch nursing home system is organized, i.e. nursing home medicine is a specific medical discipline and elderly care physicians are employed within the nursing home [25], might contribute to a holistic view of physicians on the personal dignity of the residents they care for.

### Comparison with the residents’ perspective

Comparison with the dignity-related aspects that were mentioned by the residents themselves [15] shows many similarities. Nursing home residents also mentioned treatment and attitude of nursing home staff as influential to their dignity and ascribed some experienced indignities to the scarcity of personnel. Noteworthy however was the weight of importance given to the themes mentioned. Aspects related to privacy matters were for example less prominent in the interviews with residents as compared to the interviews with staff. Especially environmental privacy was not such an issue for residents: some residents who shared a room with others even mentioned this to heighten their feelings of safety and comfort. In contrast, residents emphasized much more than nursing home staff the importance of receiving social support from relatives and society, and not feeling a burden or stigmatized, as influential to their dignity. These findings are rather similar to the results from a study of Baillie [26], who noticed that for patients in a hospital setting dignity was enhanced by having contact with fellow patients suffering the same fate, and by having a good relationship with the staff, whereas staff members were largely unaware of the beneficial effects of these relational factors, and were more focussed on privacy matters. A possible explanation for the finding that staff put less emphasis on social support and stigmatization as compared to residents, might be that these elements are for a large part outside of staff’s control, while they can exert control over safeguarding someone’s privacy and treating someone with respect. That staff already does a good job in taking care of residents’ privacy, in combination with the fact that people's preferences may change due to illness, might in addition account for the different weight given to privacy issues.

### Barriers to tailored dignity-conserving care

It is remarkable that physicians and nurses could often mention aspects that were important for a particular resident’s dignity, but, when asked about their contribution to this resident’s dignity, often came up with general aspects of dignity-conserving care (e.g. treating someone with respect and taking care of his/her privacy). Thus, despite physicians’ and nurses’ belief that giving tailored care was part of dignity-conserving care, they either did not or could not always bring this about in practice. This might partly be explained by the finding that staff members intended to treat the residents the way they want to be treated themselves. By taking this adage as a guide in daily work, staff members might project their own norms and values on all nursing home residents. This does not necessarily lead to a discrepancy in values and preferences between a nursing home staff member and a particular resident. But, when it does, this could hamper the individualized aspect of giving dignity-conserving care. Another possible explanation might be that, given the staff shortages and limited time available for each resident, physicians and nurses focus on dignity-conserving aspects they are capable of, to survive the busyness. Also, an adopted task-oriented approach and professional standards which emphasize the quality of the basic ADL care might explain this general focus of nursing home staff members [27].

### Interaction between respecting professional dignity and dignity promotion

As was concluded in a study of Gallagher [28], an inextricable link exists between how a nurse respects the dignity of others (other-regarding dignity) and how she respects her own personal and professional dignity (self-regarding dignity). As we found, in line with other studies [18,19], that nursing home staff members often experience undignified situations and moral conflicts themselves while working in the nursing home, this might have negative implications for the dignity-conserving care they give to residents. The other way around seems also to hold true: if staff members feel that they are able to give care in accordance with their intention to treat others they want to be treated themselves, their own personal and professional dignity might be enhanced, which in turn might benefit the residents. Because of this interaction between other- and self-regarding dignity, it seems important to optimize the working conditions for nursing home staff, in order to enhance the extent to which nursing home staff members feel dignified themselves while working in the nursing home. A more efficient task prioritization and organization, in combination with a decrease in amount of paperwork, and an increase in both the numbers and quality of nursing home staff, could contribute to this [29].

### Strengths and limitations of the study

By asking physicians and nurses about their view on the personal dignity of a particular resident, nursing home staff members were forced to become more concrete in their considerations of dignity-conserving aspects in their daily work, which can be seen as a strength of this study. However, a limitation of this study is that some nursing home staff members may not have known a resident very well, because the residents were recently admitted to the nursing home. Especially if a resident was physically stable, the physician was not always able to reflect elaborately on his/her dignity.

## Conclusions

This study has provided more insight into the experiences and views of physicians and nurses regarding individual nursing home residents’ dignity. Despite nursing home staff members’ knowledge of dignity-conserving aspects in general and of aspects important for an individual resident’s dignity, dignity-conserving care that is tailored to an individual appears difficult to bring about in practice. Contextual barriers within the nursing home (e.g. staffing shortages), conflicting values in daily care as well as the own preferences and values that physicians and nurses bring on and project on the situation, all seem to contribute to this phenomenon. To enhance nursing home residents’ personal dignity, a focus on the background and preferences of the individual nursing home resident is required. It is therefore important that staff members become more aware of their own norms and values, and that they realise that residents might not always want to be treated the way staff members would like to be treated themselves. In addition, more attention to solve the contextual barriers within the nursing home is needed. Hence, both policy makers and nursing home managers on the one side and nurses and physicians on the other side should cooperate to improve contextual factors and discuss conflicting values with each other when attempting to give dignity-conserving care.

## Appendix

### Details about recruitment, inclusion criteria and characteristics of previously interviewed nursing home residents

*The information below is to a large extent copied from Oosterveld-Vlug et al*. 2013 [15].

Nursing home residents were recruited from four nursing homes in the Netherlands, with help from a physician, nurse or unit manager. To obtain variation in the degree of potential factors influencing dignity, these nursing homes were selected because they varied in location (3 urban, 1 rural) and privacy conditions (3 with private rooms, 1 with shared rooms). Above that, the sampling of participants was aimed at maximizing the range of residents’ characteristics (gender, age, cultural background, religion and type of illness). Eligible residents were those who were recently admitted to a long-stay unit for residents with physical illness, and able to understand the study, give informed consent and speak comprehensibly in Dutch. Residents with severe dementia were excluded from the study because of the complex subject matter of the interviews. We also excluded residents on rehabilitation wards, whose length of stay is often short.

During the inclusion period, 53 residents were approached to participate in this study. Seventeen declined, citing they felt physically or mentally unable to participate or had plans to move to another nursing home in the near future. One resident died soon after she had received the invitation letter and a further five residents were excluded by the time of the interview, due to severe problems with hearing or speech, being sedated for palliative reasons or because they returned home. This resulted in 30 participating residents (characteristics shown in Table 2), of whom seven lived in nursing home A, eleven in B, four in C and eight in nursing home D. The respondents ranged in age between 49 to 102 years and suffered often from multi-morbidity and deterioration of bodily functions because of old age (e.g. impaired hearing/sight). Most residents had a Dutch cultural background, whereas three respondents were born in Surinam, one in Indonesia and one in Poland.

**Table 2:** Characteristics of the previously interviewed nursing home residents

**Respondent**	**Characteristics**			
	**Sex**	**Age range**	**Illness(es)**	
1	Woman	81–90	CVA, COPD, rheumatoid arthritis	
2	Man	≤ 60	CVA	
3	Woman	≤ 60	Crohn’s disease	
4	Woman	71–80	Not able to stand as a result of trauma	
5	Man	81–90	CVA, problems with kidney	
6	Woman	61–70	CVA, COPD	
7	Man	71–80	CVA	
8	Man	61–70	Multiple system atrophy	
9	Man	≤ 60	Liver cirrhosis	Terminally ill patient
10	Man	81–90	Heart failure	
11	Woman	81–90	Arthrosis	
12	Woman	> 90	Arthrosis, macula degeneration	
13	Woman	81–90	Ankle fracture, abscess	
14	Woman	81–90	Cancer of pancreas, heart failure	Terminally ill patient
15	Man	> 90	Rheumatoid arthritis	
16	Woman	81–90	CVA, heart failure	
17	Woman	81–90	Huntington’s disease	
18	Man	71–80	CVA	
19	Woman	81–90	CVA, diabetes, aneurysm of aorta	
20	Woman	61–70	Cerebral hemorrhage as a result of trauma	
21	Woman	> 90	Heart failure	
22	Man	81–90	Proximal muscle weakness	
23	Woman	71–80	Hydrocefalus (accumulation of fluid in brains)	
24	Man	81–90	Neglect of self, diabetes, not able to stand	
25	Woman	71–80	Myocardial infarction, heart failure, poliomyelitis	
26	Woman	81–90	CVA	
27	Woman	81–90	Arthrosis, osteoporosis, transient ischaemic attacks	
28	Man	71–80	Paralyzed as a result of trauma, diabetes	
29	Woman	≤ 60	Hydrocefalus as a result of an aneurysm in brains	
30	Man	81–90	CVA, heart failure	

## Competing interests

The authors declare that they have no competing interests.

## Authors’ contributions

BOP conceived of the study, and all authors participated in its design. MOV performed all interviews, coded them and drafted the manuscript. IG also coded several interviews as to ensure reliability. RP, DW and BOP regularly met with MOV and IG to discuss the interview transcripts, codes and to interpret the findings. All authors helped to draft and approved the manuscript.

## Pre-publication history

The pre-publication history for this paper can be accessed here:

http://www.biomedcentral.com/1472-6963/13/353/prepub
